# Supplementary material for: Identification of Immunogenic Antigens of Naegleria fowleri Adjuvanted by Cholera Toxin
Source: Pathogens. 2020 Jun 10;9(6):460. doi: 10.3390/pathogens9060460 (PMC7350353; doi:10.3390/pathogens9060460)
Supplement: Supplementary file 1 [file pathogens-09-00460-s001.pdf]

Table S1. Details of mass spectrometry results.

Immunogenic polypeptide bands (kDa)	Accession	Protein Description	Compared Species [(a)]	Mol. weight (Da) / Theoretical pl (pH) of complete protein	Peptide match /sequence coverage (%)
250	Q25561	Myosin II heavy chain Fragment	*N. fowleri*	87729 / 5.4126	276 / 85.6568
250	B5M	Actin Fragment	*N. fowleri*	41698 / 5.0977	77 / 83.2
250	ACT1	Actin 1	*N. fowleri*	41700 / 5.0977	77 / 36
250	NF09	Unknown protein NF009 from 2D PAGE Fragment	*N. fowleri*	2011/ 9.8833	6 / 80
250	Q94626	Cpn 60 Fragment	*N. fowleri*	38813 / 4.8545	61 / 82.5485
250	Q25558	Ubiquitin Fragment	*N. fowleri*	13418 / 9.7969	36 / 77.9661
250	Q9TWP8	Cysteine protease Fragment	*N. gruberi*	2323 / 5.3862	15 / 100
100	B5M6J9	Actin Fragment	*N. fowleri*	41698 / 5.0977	30 / 44.5333
100	Q25558	Ubiquitin Fragment	*N. fowleri*	13418 / 9.7969	9 / 52.5424
100	Q25552	Heat shock protein	*N. fowleri*	68949 / 5.4814	65 / 57.8947
100	Q95	Membrane protein	*N. fowleri*	19919 / 6.9844	19 / 53.5912
100	C6L6E3	Amino acid decarboxylase Fragment	*N. fowleri*	34492 / 6.1948	25 / 47.557
100	Q95VC2	26S proteasome subunit Fragment	*N. fowleri*	15647 / 4.9893	10 / 70.5036
100	Q25548	Penicillin amidase homolog Fragment	*N. fowleri*	66242 / 5.8081	30 / 42.6146
100	M4H5H9	ATP synthase subunit alpha	*N. fowleri*	61917 / 8.499	30 / 45.2727
100	Q25561	Myosin II heavy chain Fragment	*N. fowleri*	87729 / 5.4126	92 / 57.9088
100	Q6B	Hsp70	*N. fowleri*	71362 / 4.9614	48 / 45.3718
100	M4H5R4	Ribosomal protein S3	*N. fowleri*	63570 / 10.292	27 / 40.9709
100	M1H4M8	Putative cytosolic carboxypeptidase 6	*N. fowleri*	79105 / 8.3994	34 / 41.847
100	D2UXB7	Putative uncharacterized protein	*N. gruberi*	37129 / 5.0728	10 / 36.747
100	D2VY23	Predicted protein	*N. gruberi*	99878 / 6.8042	23 / 18.0995
100	D2W5T0	Predicted protein	*N. gruberi*	35402 / 6.769	5 / 11.6719

Table S1. Details of mass spectrometry results.

100	D2VU93	Predicted protein	*N. gruberi*	17630 / 10.2686	9 / 44.8052
100	D2VTG7	Polyubiquitin	*N. gruberi*	17338 / 7.7124	8 / 38.961
100	D2V120	Calponin homology domain protein Fragment	*N. gruberi*	37157 / 3.9111	3 / 4.3478
100	D2UXT6	Predicted protein Fragment	*N. gruberi*	19357 / 6.3735	2 / 12.7168
100	D2W142	Glyceraldehyde 3 phosphate dehydrogenase	*N. gruberi*	36576 / 6.6548	6 / 18.9189
100	D2V2V3	Glutamate dehydrogenase	*N. gruberi*	50736 / 7.3022	9 / 26.6234
100	D2VMT9	AP complex subunit beta	*N. gruberi*	101748 / 4.938	15 / 17.6149
100	D2VWQ6	Catalase	*N. gruberi*	55347 / 8.8608	6 / 22.2904
100	D2V5Y1	Dihydroorotate dehydrogenase family protein	*N. gruberi*	113167 / 5.7949	15 / 20.3276
100	D2V5T0	ATP synthase subunit beta	*N. gruberi*	65973 / 5.0054	5 / 10.3161
70	ACT1	Actin 1	*N. fowleri*	41700 / 5.0977	20 / 56.8
70	ACT2	Actin 2	*N. fowleri*	41126 / 5.0698	17 / 46.0916
70	Q95UJ2	Membrane protein	*N. fowleri*	19919 / 6.9844	22 / 52.4862
70	Q6B3P1	Hsp70	*N. fowleri*	71362 / 4.9614	/ 38.5432
70	D2UZ33	Predicted protein	*N. gruberi*	78312 / 4.9277	17 / 26.8722
70	D2VWJ8	Predicted protein	*N. gruberi*	67187 / 4.7974	10 / 22.4422
70	D2VSC1	Predicted protein	*N. gruberi*	91679 / 4.8984	14 / 21.0462
70	D2VDR3	Methylcrotonyl CoA carboxylase	*N. gruberi*	78185 / 6.5259	9 / 20.5421
70	D2V9C6	Predicted protein	*N. gruberi*	39441 / 9.7676	6 / 31.4121
70	D2VMT9	AP complex subunit beta	*N. gruberi*	101748 / 4.938	10 / 14.9891
70	D2V5V4	Clathrin heavy chain	*N. gruberi*	193131 / 5.3628	21 / 16.7258
70	D2V5T0	ATP synthase subunit beta	*N. gruberi*	65973 / 5.0054	9 / 28.2862
70	D2VGK9	Predicted protein	*N. gruberi*	75954 / 5.1768	5 / 10.6061
70	D2UXG9	Isovaleryl CoA dehydrogenase	*N. gruberi*	44317 / 6.1729	7 / 19.5545
70	D2VWE1	Predicted protein	*N. gruberi*	57586 / 4.9248	4 / 9.2184

Table S1. Details of mass spectrometry results.

50	C6L6E3	Amino acid decarboxylase Fragment	*N. fowleri*	34492 / 6.1948	39 / 57.0033
50	ACT1	Actin 1	*N. fowleri*	41700 / 5.0977	21 / 34.4
50	PFP	Pyrophosphate fructose 6 phosphate 1 phosphotransferase	*N. fowleri*	48068 / 7.374	44 / 50.3433
50	Q95UJ2	Membrane protein	*N. fowleri*	19919 / 6.9844	16 / 51.3812
50	Q25549	Thioredoxin homolog	*N. fowleri*	11172 / 4.0342	6 / 22.449
50	Q6B3P1	Hsp 70	*N. fowleri*	71362 / 4.9614	55 / 58.8771
50	Q25561	Myosin II heavy chain Fragment	*N. fowleri*	87729 / 5.4126	75 / 58.7131
50	D2V2V3	Glutamate dehydrogenase	*N. gruberi*	50736 / 7.3022	36 / 49.3507
50	D2UXB7	Putative uncharacterized protein	*N. gruberi*	37129 / 5.0728	11 / 25
50	D2VT95	Predicted protein	*N. gruberi*	52935 / 6.627	10 / 18.8312
50	D2VWF2	Predicted protein	*N. gruberi*	52059 / 5.6514	6 / 11.2554
50	D2V5S2	Pyrophosphate fructose 6 phosphate	*N. gruberi*	46724 / 6.8262	7 / 9.8592
50	D2V0Z9	Predicted protein	*N. gruberi*	46089 / 4.6523	3 / 8.9776
50	D2W1E4	Adenosylhomocysteinase	*N. gruberi*	51769 / 5.439	7 / 14.3763
50	D2VLM1	Coronin	*N. gruberi*	50988 / 6.186	11 / 31.5098
50	D2V3J1	Elongation factor Tu	*N. gruberi*	48997 / 6.9536	6 / 18.3445
50	D2VV89	Predicted protein	*N. gruberi*	53638 / 4.8853	6 / 11.1842
50	D2V5T0	ATP synthase subunit beta	*N. gruberi*	65973 / 5.0054	7 / 17.6373
50	D2V9Y7	Predicted protein	*N. gruberi*	50230 / 6.1963	3 / 5.4422
50	D2V3R0	Alanine aminotransferase	*N. gruberi*	55072 / 6.5112	7 / 17.0732
50	D2W0R2	Predicted protein	*N. gruberi*	43123 / 4.3022	8 / 16.8901
50	D2W1W8	Predicted protein	*N. gruberi*	48054 / 5.1885	6 / 15.6542
50	Q2MM01	Elongation factor 1 alpha Fragment	*N. gruberi*	44557 / 7.9043	5 / 11.6915
50	D2VCZ2	Protein disulfide isomerase	*N. gruberi*	52822 / 4.6025	7 / 15.5462
50	D2UYA5	Serine hydroxymethyltransferase	*N. gruberi*	55555 / 9.1128	7 / 20.5589
50	D2V193	Rab GDP dissociation inhibitor	*N. gruberi*	49867/ 5.8359	8 / 14.6396

Table S1. Details of mass spectrometry results.

50	D2V7J7	Actin related protein ARP3	*N. gruberi*	47936 / 5.4287	2 / 5.9242
50	D2VJF9	RhoGEF domain containing protein	*N. gruberi*	101687 / 8.7598	8 / 8.8268
50	D2UXY9	Predicted protein	*N. gruberi*	50248 / 6.7661	10 / 38.0313
50	D2VP45	Succinate CoA ligase ADP forming subunit beta mitochondrial	*N. gruberi*	46855 / 5.5693	5 / 11.8605
50	D2UY50	4 aminobutyrate aminotransferase	*N. gruberi*	49745 / 6.6343	11 / 24.3182
50	D2W3B8	Predicted protein	*N. gruberi*	22492 / 5.8125	4 / 13.4021
50	D2V1E8	Dihydrolipoamide succinyltransferase	*N. gruberi*	39705 / 6.4146	6 / 20.5962
50	D2VB42	Predicted protein	*N. gruberi*	25026 / 6.4175	4 / 24.6512
50	D2VBF6	Predicted protein	*N. gruberi*	44840 / 5.647	9 / 24.937
50	D2VLT8	Predicted protein	*N. gruberi*	29111 / 7.2202	3 / 14.5669
50	D2UZW5	Predicted protein	*N. gruberi*	49359 / 5.5151	5 / 9.0487
50	D2W2M7	Predicted protein	*N. gruberi*	63894 / 5.7993	7 / 15.3005
37	ACT2	Actin 2 Fragment	*N. fowleri*	41126 / 5.0698	72 / 63.8814
37	B5M6J9	Actin Fragment	*N. fowleri*	41698 / 5.0977	98 / 70.9333
37	M4H5H9	ATP synthase subunit alpha	*N. fowleri*	61917 / 8.499	29 / 36
37	Q25559	Ubiquitin Fragment	*N. fowleri*	13658 / 5.7847	11 / 66.1157
37	Q25561	Myosin II heavy chain Fragment	*N. fowleri*	87729 / 5.4126	78 / 69.8391
37	D2UXB7	Putative uncharacterized protein	*N. gruberi*	37129 / 5.0728	38 / 51.506
37	D2VE39	Putative uncharacterized protein	*N. gruberi*	41849 / 4.8911	26 / 46.6844
37	D2VZJ8	Putative uncharacterized protein	*N. gruberi*	21849 / 5.3789	20 / 52.0202
37	D2VUS6	Conventional actin	*N. gruberi*	41669 / 5.2148	16 / 27.0053
37	D2W142	Glyceraldehyde 3 phosphate dehydrogenase	*N. gruberi*	36576 / 6.6548	10 / 20.4204
37	D2V8I2	Putative uncharacterized protein	*N. gruberi*	42274 / 5.0508	14 / 19.5767
37	D2VM36	Predicted protein	*N. gruberi*	35625 / 8.6938	9 / 25.9146
37	D2VKN7	Predicted protein	*N. gruberi*	40558 / 11.7422	3 / 6.8493
37	D2VK26	Mitogen activated protein kinase	*N. gruberi*	40951 / 8.1885	3 / 29

Table S1. Details of mass spectrometry results.

37	D2VSP0	Predicted protein	*N. gruberi*	39962 / 4.4678	9 / 14.4092
37	D2VZB1	Malate dehydrogenase	*N. gruberi*	35199 / 9.2959	9 / 26.2195
37	D2W2C5	Prohibitin	*N. gruberi*	33787 / 9.7046	9 / 24.5098
37	D2VQ70	Isocitrate dehydrogenase NAD subunit mitochondrial	*N. gruberi*	39096 / 8.1489	6 / 13.0194
37	D2VRD7	Isocitrate dehydrogenase NADP dependent	*N. gruberi*	36836 / 7.7446	14 / 32
37	D2VNA9	Mitochondrial trans-2 enoyl CoA reductase	*N. gruberi*	40181 / 9.1421	6 / 7.7135
37	D2UY23	Inosine 5 monophosphate dehydrogenase	*N. gruberi*	37515 / 7.7446	6 / 20.2312
37	D2V5T0	ATP synthase subunit beta	*N. gruberi*	65973 / 5.0054	8 / 19.1348
37	D2VKI4	Predicted protein	*N. gruberi*	39031 / 8.9106	7 / 23.2836
37	D2V115	ARF SAR family small GTPase	*N. gruberi*	40269 / 5.0186	5 / 16.0458
37	D2VD78	Jun kinase activation domain binding protein	*N. gruberi*	37764 / 5.5459	4 / 6.0423
37	D2VRL5	Predicted protein	*N. gruberi*	33547 / 5.9033	1 / 4.2345
37	Q2MM01	Elongation factor 1 alpha Fragment	*N. gruberi*	44557 / 7.9043	4 / 12.4378
37	D2V803	NAD dependent epimerase dehydratase family protein	*N. gruberi*	36787 / 5.9897	4 / 9.7264
37	D2VEC2	Putative uncharacterized protein	*N. gruberi*	42428 / 4.9966	12 / 22.0472
19	Q95UJ2	Membrane protein	*N. fowleri*	19919 / 6.9844	84 / 66.8508
19	NF16	Unknown protein NF016 from 2D PAGE Fragment	*N. fowleri*	2575 / 7.8486	5 / 73.913
19	Q9NAY9	Calcineurin B	*N. fowleri*	21674 / 4.374	17 / 41.0526
19	Q27706	Fructose 1 6 bisphosphatase homolog Fragment	*N. fowleri*	19719 / 9.4453	23 / 70.3911
19	W0SLB1	Photoactivated adenylyl cyclase	*N. fowleri*	43378 / 5.666	42 / 58.0729
19	D2VL67	40S ribosomal protein S13	*N. gruberi*	17121/ 10.5454	8 / 48.0263
